# Development of MoS_2_ Modified SPE Based Electrochemical Immunosensors Sandwiched by Au NP Labeled Antibodies for Detecting *Bovine rotavirus* in Calves

**DOI:** 10.3390/life16030464

**Published:** 2026-03-12

**Authors:** Ayşenur Akkaya, Derya Bal Altuntaş, Chao Zhang, Sema Aslan, Aziz Kerim Çelik, Berkan Karagöz, Ümmünur Çelik, İbrahim Sözdutmaz, Ramin Jahangirov

**Affiliations:** 1 Department of Chemistry, Muğla Sıtkı Kocman University, Muğla 48000, Türkiye; aysenurakkaya13@gmail.com (A.A.); sozcan87@gmail.com (S.A.); azizkerimcelik@gmail.com (A.K.Ç.); krgz.berkan@gmail.com (B.K.); ummunurcelik@gmail.com (Ü.Ç.); 2 Department of Bioengineering, Faculty of Engineering and Architecture, Recep Tayyip Erdogan University, Rize 53100, Türkiye; 3 Department of Neurosurgery, Zhujiang Hospital, Southern Medical University, Guangzhou 510282, China; czhangsinap@163.com; 4 Department of Preclinical Sciences, Faculty of Veterinary Medicine, Erciyes University, Kayseri 38000, Türkiye; isozdutmaz@erciyes.edu.tr; 5 Department of Renewable Energy Sources and Technologies, Muğla Sıtkı Kocman University, Muğla 48000, Türkiye; ramin.cahangir2@gmail.com

**Keywords:** electrochemical biosensor, immunosensor, *Bovine rotavirus*, MoS_2_ nanoparticles, screen-printed carbon electrodes

## Abstract

*Bovine rotavirus* (BRV) is one of the leading causes of neonatal diarrhea in calves and remains a major concern in veterinary medicine due to its high morbidity and economic impact. Rapid, sensitive, and cost-effective diagnostic approaches are therefore required for early detection and disease control. In this study, electrochemical immunosensors were developed for the detection of BRV with the aim of improving existing multiplex diagnostic strategies. Screen-printed carbon electrodes (SPEs) were employed as the sensing platform and modified with molybdenum disulfide nanoparticles (MoS_2_ NPs) to enhance electrochemical performance. Mouse monoclonal antibodies against the BRV VP6 protein were immobilized onto the electrode surface, followed by blocking with bovine serum albumin. BRV detection was carried out using differential pulse voltammetry, cyclic voltammetry, and electrochemical impedance spectroscopy. To further improve sensitivity, a sandwich immunoassay format was constructed using gold nanoparticle-labeled secondary antibodies. The MoS_2_-modified sandwich immunosensor exhibited superior analytical performance, achieving a limit of detection of 1.11 ng/mL, a limit of quantification of 3.72 ng/mL, a relative standard deviation of 1.89% (*n* = 5), and a linear response with R^2^ = 0.99. The developed immunosensors demonstrated reliable performance in real sample analysis, with a selectivity rate of 100 ± 2.95%. These findings suggest that MoS_2_-based electrochemical immunosensors offer a promising platform for rapid and sensitive BRV detection and have potential applications in veterinary diagnostics.

## 1. Introduction

Pathogens such as *Bovine rotavirus* (BRV), *Escherichia coli* (*E. coli*), *Bovine coronavirus* (BCoV), and *Cryptosporidium parvum* cause significant economic and health challenges, particularly in calves, due to their high mortality rates. Although traditional diagnostic methods are effective in terms of sensitivity and accuracy, they are often costly, time-consuming, and reliant on laboratory settings. *Bovine rotavirus* (BRV) is a major viral pathogen responsible for neonatal diarrhea in calves, leading to significant economic losses in the livestock industry. Rapid and accurate detection of BRV, particularly under field conditions, is essential for preventing disease transmission and enabling early treatment. While conventional diagnostic methods such as ELISA, Western blot, SDS-PAGE, and PCR provide high sensitivity and specificity, they suffer from critical limitations, namely high cost, long processing times, reliance on well-equipped laboratories, and the need for trained personnel.

This dependency highlights the growing need for rapid, portable, and cost-effective diagnostic methods, particularly for field applications [1,2].

Previous studies have developed a fully domestic multiplex diagnostic kit using locally produced antibodies for the on-site detection of BRV, *E. coli*, BCoV, and *C. parvum*, which cause mass mortality in calves. The detection and validation methods employed in these studies included Enzyme-Linked Immunosorbent Assay (ELISA) [3], Western Immunoblotting (Western blot) [4], Sodium Dodecyl Sulfate–Polyacrylamide Gel Electrophoresis (SDS-PAGE) [5], and Polymerase Chain Reaction (PCR) [6].

Techniques, which are commonly used in standard serological diagnostic kit applications [7]. However, it has been observed that electrochemical methods, which can provide practical and highly sensitive results, were not utilized during the validation phase. The potential to adapt the diagnostic kit for alternative applications and transform it into an electrochemical diagnostic kit is highly promising [8].

In this regard, conducting parallel experiments using the same materials as the diagnostic kit on both bare and modified electrodes via electrochemical methods is deemed highly valuable. Previous studies have reported successful applications of electrochemical methods for detecting various viruses [9,10]. Electrochemical methods are widely preferred in sensor and biosensor applications due to their numerous advantages over traditional methods, such as not requiring complex conditions, minimal material usage, short measurement times, cost-effectiveness, and the lack of need for highly skilled personnel [11]. An electrochemical immuno-cytosensor modified with nanofibers for the determination of carcinoembryonic antigen [12,13,14].

A review of the literature revealed that previous electrochemical studies on BRV primarily focused on characterizing the loading properties of the VP6 protein but did not examine it as an analytical biosensor [15]. This study conducted a complete biosensor application. Notably, the immunosensor aspect of this study, developed using molybdenum disulfide nanoparticles (MoS_2_ NP)-enhanced screen-printed carbon electrodes (SPE), is not present in the existing literature.

VP6 is the major structural protein of *Rotavirus*, forming the middle layer of the viral triple-layered particle. It is highly conserved across *Rotavirus* species and is the most abundant viral protein, making it an ideal target for immunological detection methods. The VP6 antigen is commonly used in diagnostic assays due to its stability and high immunogenicity [16,17,18,19].

The novelty of this work lies in the development of a dual-enhanced sandwich immunosensor architecture that combines MoS_2_-modified SPE for improved electron transfer and antibody immobilization with AuNP-labeled secondary antibodies for signal amplification. While VP6 protein has been used as a target in previous BRV detection studies, this specific electrochemical sandwich configuration has not been previously reported.

The analyses performed in this study are expected to pave the way for electrochemical applications of the developed domestic diagnostic kit for other viruses. Upon completing the processes for all viruses, the current system can also be adapted into a more sensitive electrochemical diagnostic kit. Compared to ELISA, Western blot, SDS-PAGE, PCR methods, electrochemical techniques have been reported to exhibit much higher sensitivity in numerous biosensor applications [20]. The absence of electrochemical validation methods in this context is considered a limitation, and incorporating such methods is expected to provide significant technological contributions to further enhance the study.

In recent years, electrochemical biosensors have emerged as a promising alternative due to their advantages, including high sensitivity, speed, and low material requirements. These technologies stand out not only for their cost-effectiveness but also for their minimal need for complex sample preparation processes [21,22]. Specifically, the use of MoS_2_ NPs holds great promise for optimizing biosensor performance by increasing surface area and electrochemical activity [23,24].

In the literature, most studies on the VP6 protein have focused on its characterization rather than its application as an analytical component in electrochemical biosensors. This gap indicates the potential for biosensor technology to offer a broader application range compared to traditional methods [25].

This study aims to address this knowledge gap by developing and evaluating electrochemical immunosensors using both bare and MoS_2_ NP-modified SPEs for the detection of BRV. The immunosensors were designed to exploit the advantages of electrochemical methods, including high sensitivity, short measurement times, and minimal material usage, while overcoming limitations of traditional diagnostic approaches such as ELISA and PCR [23].

A stepwise schematic of the immunosensor fabrication process is illustrated in upcoming sections to enhance clarity in the sensor development stages. The dual-nanomaterial strategy employs MoS_2_ and AuNPs in complementary, non-redundant roles. MoS_2_ modification of the electrode surface enhances (1) surface area for increased antibody loading, (2) electron transfer kinetics through its semiconducting properties, and (3) antibody immobilization stability through favorable surface chemistry. In contrast, AuNP-labeled secondary antibodies provide post-binding signal amplification through (1) increased electroactive surface area, (2) enhanced electron transfer to the redox mediator, and (3) sandwich complex formation. The synergistic combination of these two nanomaterials at different stages of the sensing mechanism results in superior analytical performance compared to single-nanomaterial systems, as demonstrated in our comparative results. The incorporation of MoS_2_ NP was specifically targeted to enhance electrode surface area and electrochemical conductivity, enabling improved antigen–antibody binding and detection limits. Analytical parameters such as the limit of detection (LOD), limit of quantification (LOQ), linear range, and relative standard deviation (RSD) were optimized to ensure reliable performance.

## 2. Experimental Sections

### 2.1. Chemical Substances and Measurements

MoS_2_ NP (99.5%), phosphate-buffered saline (PBS), potassium ferrocyanide (K_4_Fe(CN)_6_·3H_2_O), potassium hexacyanoferrate (K_3_Fe(CN)_6_), potassium chloride (KCl), sodium hydroxide (NaOH) (98.00%), HCl (analytical pure) were purchased from Sigma-Aldrich (https://www.sigmaaldrich.com, Darmstadt, Germany), monoclonal anti-VP6 mAb antibodies (VP6mAb), bovine serum albumin (BSA), and BRV were supplied as a gift from Erciyes University Teknopark, Klonbiyotek (Kayseri, Türkiye). BRV concentrations are given as VP6 protein concentration (ng/mL) determined by Bradford assay, as the immunosensor specifically detects the VP6 antigen.

Cyclic voltammetry (CV), electrochemical impedance spectroscopy (EIS), and differential pulse voltammetry (DPV) electrochemical measurements were performed using the Metrohm DropSens DRP-STAT-I400 Potentiostat/Galvanostat (Llanera (Asturias), Spain) electrochemical workstation. The electrochemical studies were conducted with a three-electrode system of carbon-based screen-printed electrodes (SPE) (Eco Chemie, Utrecht, The Netherlands). The morphological analysis images of the structures were obtained using a Zeiss Sigma 300 scanning electron microscope (SEM–EDS) (Baden-Württemberg, Germany).

Three complementary electrochemical techniques were employed, each serving a distinct analytical purpose: (1) Cyclic Voltammetry (CV) was used to characterize the electrochemical behavior of the modified electrodes and to confirm successful stepwise modification through changes in peak current and potential. (2) Electrochemical Impedance Spectroscopy (EIS) was employed to monitor interfacial changes during electrode modification and biomolecular binding events through charge transfer resistance measurements. (3) Differential Pulse Voltammetry (DPV) was selected as the primary quantitative detection technique due to its superior sensitivity and lower background current, making it ideal for low-concentration analyte detection. CV and EIS serve as characterization and validation tools, while DPV provides the analytical signal for quantification.

### 2.2. Modification of SPEs with MoS_2_ NPs

In the initial stage, the electrochemical differences between bare SPE and MoS_2_ NP-modified SPEs were investigated. The goal was to determine the optimal MoS_2_ NP concentration for modification. SPEs were modified using varying amounts of MoS_2_ NPs (2, 3, 5, 7, and 10 mg/mL) dissolved in PBS. The prepared solutions were drop-cast onto the carbon-printed working electrode of the SPE and allowed to dry.

**Figure 1:**
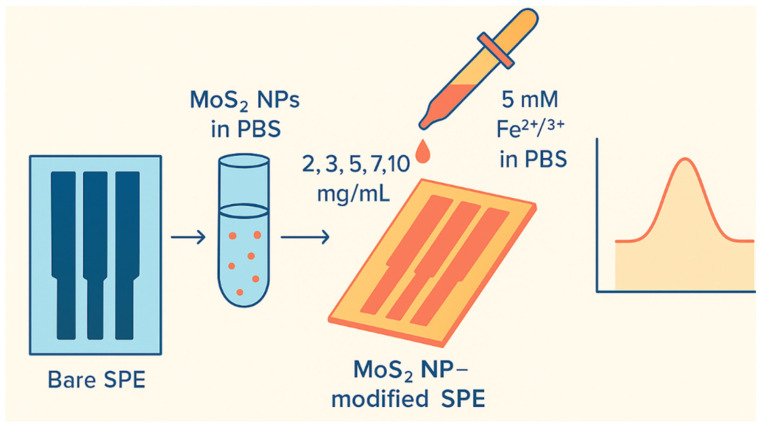
Modification of the SPE electrode with MoS_2_ NP and the representative measurement graph.

The resulting modified electrodes were tested by drop-casting 20 µL of PBS solution containing a 5 mM Fe^2+^/^3+^ redox couple onto the electrode surface (Figure 1). Current responses obtained from these measurements were analyzed and compared to those from bare SPEs. Following the protocol of [26], MoS_2_ and AuNPs were co-dispersed via ultrasound in acetic acid prior to electrode modification, ensuring a uniform nanocomposite layer.

### 2.3. Immunosensor Development Studies with SPE and SPE/MoS_2_ Electrodes

For the conversion of electrodes into immunosensors and the determination of VP6 protein, the first step involved the modification of the electrodes using mouse-derived monoclonal VP6mAb. Suspensions containing 10 ng/mL of VP6mAb were drop-cast onto the surface of SPE and SPE/MoS_2_ electrodes.

**Figure 2:**
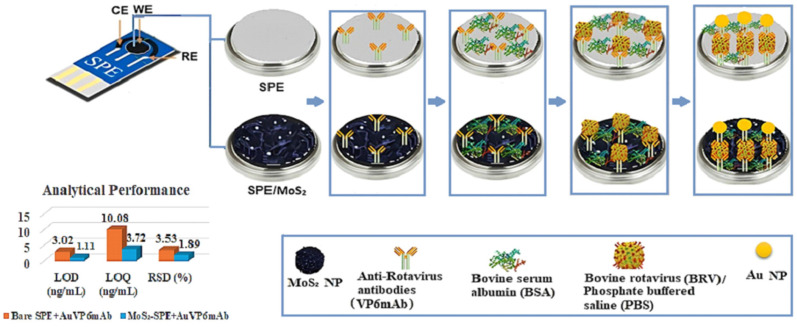
Illustrates the fabrication steps and measurement procedures scheme of the immunosensor stepwise.

Subsequently, any areas exposed on the electrode surface were blocked with 5% BSA to prevent non-specific binding, and immunosensors were created. Finally, an appropriate pH PBS suspension containing 10 ng/mL of the deactivated BRV was drop cast onto the electrode surface. Gold nanoparticle-labeled antibodies (AuVP6mAb) were incorporated to enhance the sensitivity of antigen–antibody binding and enable measurements at lower concentrations [27], Figure 2. The changes in the current responses due to the binding interactions were examined. The work carried out up to this point was aimed at verifying whether the bindings occurred in a proper manner. The sensor fabrication process begins with screen-printed electrodes (SPE), followed by modification with molybdenum disulfide nanoparticles (MoS_2_ NPs). Anti-BRV VP6 monoclonal antibodies are immobilized on the surface, and non-specific binding sites are blocked using bovine serum albumin (BSA). Subsequently, the sensor is exposed to BRV antigens in PBS. To enhance signal sensitivity, gold nanoparticle (Au NP)-labeled antibodies are introduced, forming a sandwich-type immunosensor configuration.

## 3. Results and Discussion

### 3.1. Optimization of the Amount of MoS_2_ NPs

For the quantity optimization of MoS_2_ nanoparticles added to SPEs, only the DPV method was employed. The potential range for the responses obtained from both bare and modified electrodes was set between −0.3 V and +0.3 V, with a scan rate of 50 mV/s.

In the quantity optimization process, the value for the 10 mg/mL suspension addition is not very precise because the MoS_2_ NP overflows from the surface. However, since the measurement was taken, it has been included in the Supplementary Document. Figures S1 and S2 (Supporting Information) show the DPV optimization of MoS_2_ NP concentrations.

### 3.2. Electrochemical Optimizations

**Figure 3:**
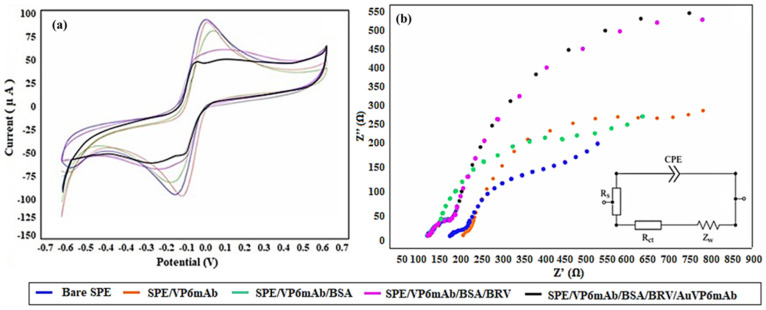
(**a**) CV characterization of the fabrication stages of the immunosensor developed for BRV determination based on SPE. (**b**) EIS characterization spectra for each stage of the SPE-based BRV immunosensor (5 mM [Fe(CN)_6_]^3−/4−^) in 50 mM PBS (pH 7.4), −0.7 V to +0.7 V, carried out at a scanning rate of 100 mV/s, frequency: 10^4^ to 10^−1^ Hz.

The CV measurements of the modifications on the SPE electrode are presented in Figure 3a, while the EIS measurements are shown in Figure 3b. In CV measurements, the current value obtained with the bare SPE electrode decreases after each binding, as the electroactive sites on the electrode surface progressively close [28]. In line with these results, the increase in impedance after binding has been confirmed by the rise in the semi-circles of the impedance spectrum in Figure 3b, due to the gradual increase in resistance on the electrode surface. The Rct values corresponding to the semi-circles are provided in Table 1. These measurements are initial measurements. A series of optimizations have been performed in subsequent studies to determine the optimal binding conditions.

The measurements obtained before and after the binding with BRV were compared by taking the difference in currents from the voltammograms. Optimization graphs, constructed using the voltammograms and the current responses obtained from them, are provided sequentially. For this purpose, DPV measurements were performed. The binding at the value where the highest current difference was obtained was considered the highest, and this value was determined as optimal. After the optimum parameters were sequentially determined, the optimization of other parameters was carried out with this value kept constant. Analytical characteristics and subsequent studies were conducted under fully optimized conditions.

### 3.3. Optimization Procedure of the Immunosensor

#### 3.3.1. pH Optimization

To mimic conditions closest to the virus binding environment, PBS solutions with pH values ranging around 7.4, which is close to the physiological pH, were prepared [29]. BRV solutions were prepared in these PBS solutions and applied onto the immunosensor, followed by DPV measurements. PBS solutions were prepared at pH values of 6, 6.5, 7, 7.5, and 8, and BRV solutions with a concentration of 10 ng/mL were prepared in microcentrifuge tubes using these PBS solutions. While pH 7.4 showed optimal performance, it should be noted that pH adjustments with HCl and NaOH may alter solution conductivity and ionic strength, potentially contributing to the observed electrochemical differences alongside pH-dependent antibody–antigen binding affinity. Future studies using constant ionic strength buffers could further clarify the specific pH effect. The DPV voltammograms and the corresponding current response graph are presented in Figures S3 and S4.

#### 3.3.2. Temperature Optimization

Similarly, to determine the optimal temperature, a temperature range around the physiological temperature of 36 °C was selected, including 24, 28, 32, 36, and 40 °C. PBS solutions at pH 7.4 were prepared and heated in water baths set to 24, 28, 32, 36, and 40 °C, and 10 ng/mL R solutions were applied to the electrode surface at each temperature. The optimal binding temperature was determined based on these measurements. The DPV voltammograms obtained are shown in Figure S5, and the optimization graph derived from these voltammograms is presented in Figure S6. Temperature optimization showed that the immunosensor performs efficiently at both room temperature (25 °C) and 37 °C (current values showed just a 0.5 mA difference; Figure S6) with comparable signal intensities. This finding is practically significant, as it demonstrates the sensor’s functionality at room temperature, eliminating the need for temperature control in field applications. Nevertheless, because the physiological temperature is 37 °C, this temperature was selected, and subsequent studies were continued [29].

#### 3.3.3. Incubation Time Optimization

Finally, to determine the optimal incubation time, solutions at pH 7.4 and 36 °C were maintained, and the electrode was placed in a saturated vapor environment of this solution for incubation times of 20, 25, 30, 33, and 35 min. During this stage, the non-bound BRV antigens were washed off the electrode surface with PBS, and DPV measurements were taken. At this stage, the optimum duration was determined to be 30 min. After this step, all optimizations were completed.

The obtained voltammograms are shown in Figure S7, and the optimization graph derived from these voltammograms displaying current values is shown in Figure S8.

### 3.4. Structural Characterization

**Figure 4:**
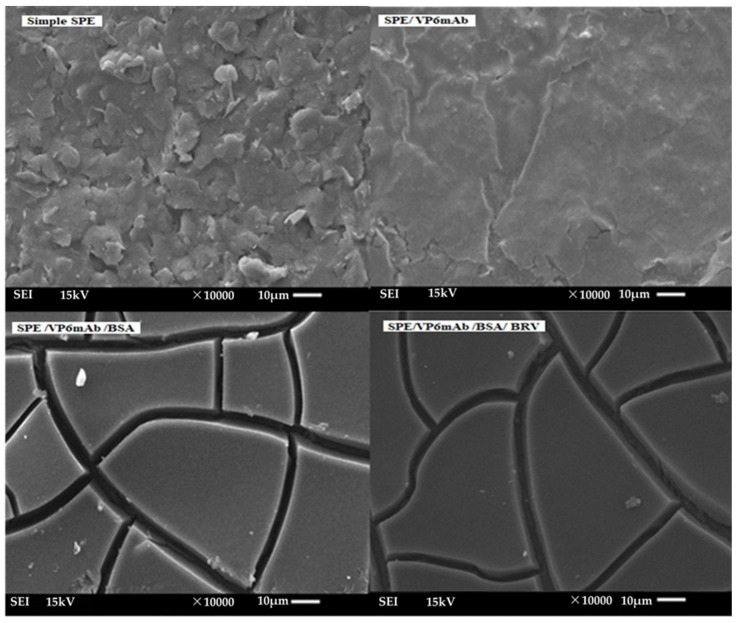
SEM images of the immunosensor production stages at ×10,000 magnification.

The morphological variations in the bindings on the SPE electrode are presented in Figure 4 through SEM measurements. The results clearly reflect the morphological changes caused by the bindings at each stage of the modifications. These findings are further supported by electrochemical measurements.

Figure 4 presents SEM images of the immunosensor production stages at different magnifications. First, the Simple SPE (unmodified electrode) surface, at a magnification of ×10,000, displays an irregular and natural structure with no significant modifications observed. Next, in the SPE/VP6mAb (specific antibody modification) stage, the surface appears more homogeneous, with the antibodies successfully bound to the electrode surface. A finer and more orderly structure becomes evident at this stage. In the SPE/VP6mAb/BSA modification, the addition of the BSA blocking agent over the antibodies results in a smoother, more uniform surface. BSA is homogeneously distributed across the surface, ensuring a strong binding of the antibodies. Finally, in SPE/VP6mAb/BSA/BRV modification, receptor binding leads to a more complex structure, with intensively localized bindings observed across the surface. This stage plays a crucial role in creating the active surface area of the immunosensor. The SEM images at the magnificatio of ×10,000 clearly show significant morphological changes at each modification stage. These findings validate the successful modifications to the electrode surface and confirm the evolving structural characteristics of the immunosensor. In line with [26], who reported Mo, S, and Au signals in their MoS_2_/AuNP nanocomposite by EDS, our modified SPE also shows comparable elemental features, confirming the successful deposition of both nanomaterials.

### 3.5. Formation of the Sandwich Structure and Electrochemical Optimizations for SPE/VP6mAb/BSA

In this study, calibration curves for binding to the BRV antigen, followed by gold nanoparticle-labeled VP6 antibody, were created. Initially, the current responses of the blanks were measured before antigen binding (if LOD and LOQ for AuVP6mAb were being measured, the same measurement was taken with BRV-bound immunosensors). For this, 10 measurements were taken using the SPE/VP6mAb/BSA and SPE/MoS_2_ NP/VP6mAb/BSA immunosensors. The standard deviation (Sb) and the slope (m) obtained from the calibration graph were used to calculate the LOD and LOQ as follows: LOD = (3Sb)/m and LOQ = LOD10/3. The LOD and LOQ values for the SPE/VP6mAb/BSA were found to be 1.66 ng/mL and 5.53 ng/mL, respectively.

**Figure 5:**
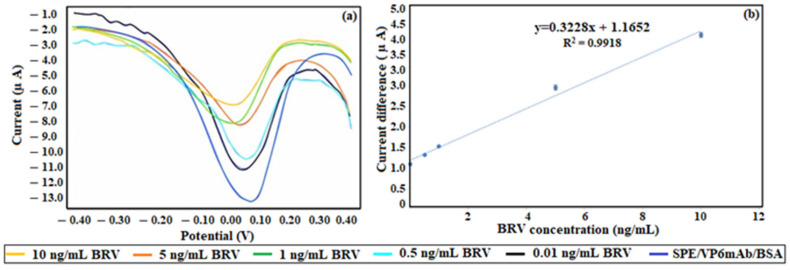
(**a**) Voltammograms were obtained for BRV determination using the SPE/VP6mAb/BSA immunosensor. (**b**) Calibration graph created from the current responses obtained after binding solutions containing R at different concentrations using SPE/RmAb/BSA (*n* = 5).

The DPV voltammograms for the BRV binding measurements of the SPE/VP6mAb/BSA immunosensor were recorded with BRV concentrations of 0.01, 0.5, 1.0, 5.0, and 10.0 ng/mL. The calibration curve derived from these voltammograms is shown in Figure 5a, while the calibration graph is provided in Figure 5b. The linear range of the calibration curve for the SPE/VP6mAb/BSA was found to be from 0.01 to 10 ng/mL. The calibration equation was determined as y = 0.3228x + 1.1652, with a correlation coefficient (R^2^) of 0.9918. RSD was calculated to be 3.49 for *n* = 5 using the equation (s/x^−^) × 100.

**Figure 6:**
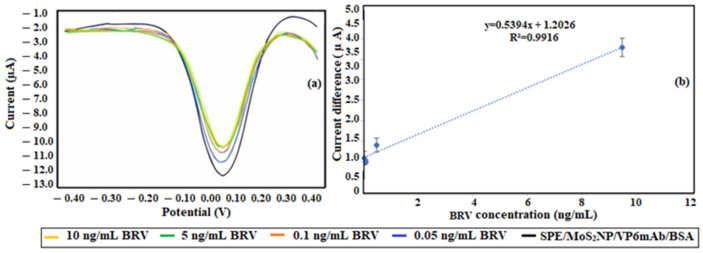
(**a**) DPV voltammograms of BRV binding to the SPE/MoS_2_NP/VP6mAb/BSA immunosensor. (**b**) Calibration graph for the binding of BRV to the SPE/MoS_2_NP/VP6mAb/BSA immunosensor. Linear range concentrations of BRV: 0.05, 0.1, 0.5, and 10 ng/mL in 50 mM PBS (pH 7.4) including 5 mM [Fe(CN)_6_]^3−/4−^, in the range of −0.3 V to +0.3 V with a scanning rate of 50 mV/s (*n* = 5).

Studies have been conducted on the virus binding using MoS_2_ NP modification. In this phase, analyses were performed using the immunosensor with bare SPE. Electrochemical measurements monitoring the binding of BRV to the SPE/MoS_2_NP/VP6mAb/BSA immunosensor are presented in Figure 6a. The current values observed in these voltammograms are shown in Figure 6b as a calibration graph.

In the formulation developed for the MoS_2_ NP-modified immunosensor for BRV determination, the LOD value was determined as 0.95 ng/mL and the LOQ value as 3.18 ng/mL. The calibration equation derived from the linear range consisting of concentrations of 0.05, 0.1, 0.5, and 10 ng/mL was found to be y = 0.5394x + 1.2026, with an R^2^ value of 0.99. The RSD value for the immunosensor at this stage, for 3 measurements, was calculated as 5.83.

With the NP modification, a decrease in both the LOD and LOQ values was observed. This indicates an improvement in the response from the immunosensor. However, it was noted that both the linear range and the RSD values still require further optimization.

**Figure 7:**
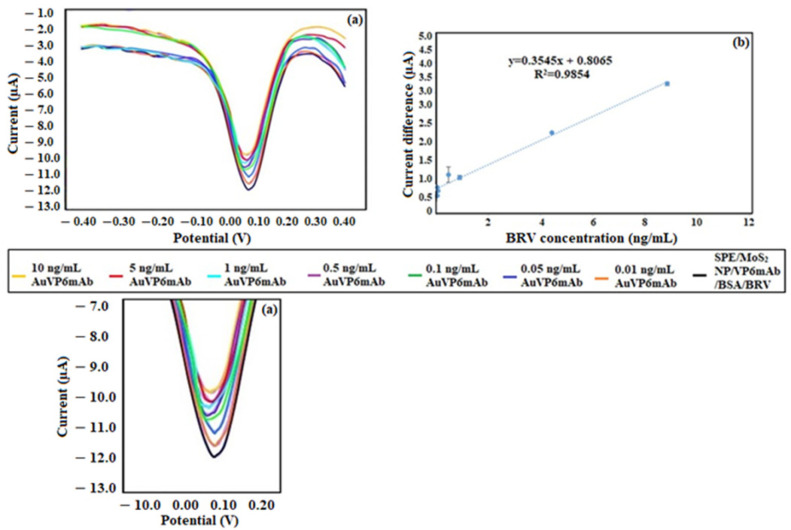
(**a**) Comparative DPV voltammograms for the binding of AuVP6mAb to SPE/VP6mAb/BSA/BRV. (**b**) Calibration curve for AuVP6mAb binding to SPE/VP6mAb/BSA/BRV. Linear range concentrations of VP6 protein: 0.01, 0.05, 0.1, 0.5, 1.0, 5.0 and 10 ng/mL in 50 mM PBS (pH 7.4) including 5 mM [Fe(CN)_6_]^3−/4−^, in the range of −0.3 V to +0.3 V with a scanning rate of 50 mV/s (*n* = 5).

After completing the studies on the binding of the R antigen, experiments to develop the sandwich structure with gold-labeled antibody (AuVP6mAb) were initiated. In these experiments, the SPE-based SPE/VP6mAb/BSA/VP6 immunosensor was treated with solutions containing AuVP6mAb at different concentrations, and the resulting analytical characteristics were presented. For this purpose, the linear range was examined from concentrations of 0.01, 0.05, 0.1, 0.5, 1, 5, and 10 ng/mL, and these concentrations were utilized in the calibration graph. The comparative display of the obtained DPV graphs is shown in Figure 7a, and the resulting calibration graph is presented in Figure 7b.

The LOD value obtained from measurements of AuVP6mAb binding to SPE/VP6mAb/BSA/BRV was calculated as 3.02 ng/mL, and the LOQ value was determined as 10.08 ng/mL. The calibration equation at this stage was found to be y = 0.3545x + 0.8065, with R^2^ = 0.99. The linear range at this stage was between 0.01 and 10 ng/mL. The developed immunosensor achieved an LOD of 1.11 ng/mL, which is 3-fold lower than conventional ELISA methods (3–5 ng/mL) and comparable to PCR-based methods, while requiring significantly shorter assay time (45 min vs. 4–6 h).

**Figure 8:**
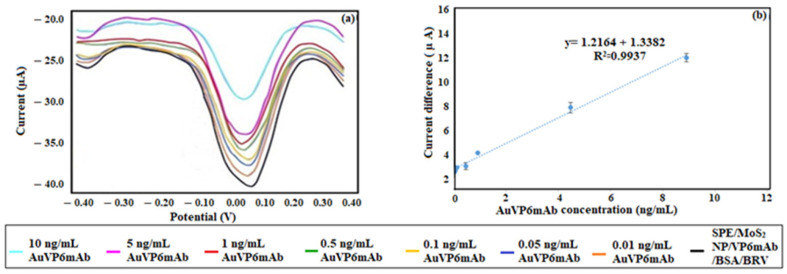
(**a**) DPV voltammograms of the interactions between SPE/MoS_2_ NP/VP6mAb/BSA/BRV and AuVP6mAb. (**b**) Calibration curve for the interactions between SPE/MoS_2_ NP/VP6mAb/BSA/BRV and AuVP6mAb (*n* = 5).

In the next stage, similar measurements were performed using the MoS_2_ NP-modified immunosensor. Measurements were conducted at the same concentrations and within the same linear range. Direct comparisons of LOD values are only made with studies targeting the same antigen (BRV or VP6 protein), while comparisons with other targets are presented solely for methodological reference. The LOD and LOQ values were found to be 1.11 ng/mL and 3.72 ng/mL, respectively. These results indicate that the binding between SPE/MoS_2_ NP/VP6mAb/BSA/BRV and AuVP6mAb benefits from the NP modification, facilitating improved electrochemical conductivity and allowing the reaction to occur at lower concentrations. For the same measurement, the RSD value was calculated as 1.89 for *n* = 5. The DPV voltammograms from the measurements are shown in Figure 8a, and the calibration curve is presented in Figure 8b. The calibration equation was determined to be y = 1.2164x + 1.3382, with R^2^ = 0.99.

### 3.6. Repeatability, Stability, Selectivity, and Real Sample Application Studies

For repeatability experiments, RSD values of the immunosensors developed under optimal conditions are presented. For stability, the measurements were repeated until a 5% current difference occurred compared to the initial measurement for SPE/VP6mAb/BSA/BRV/AuVP6mAb electrodes. The measurements were taken at 30 min, 1, 3, 6, 12, 24, and 48 h, and subsequent days until the current response difference reached 5% from the initial measurement. After 72 h, the measurement difference for BRV binding was found to be 5.12 ± 0.15%.

**Figure 9:**
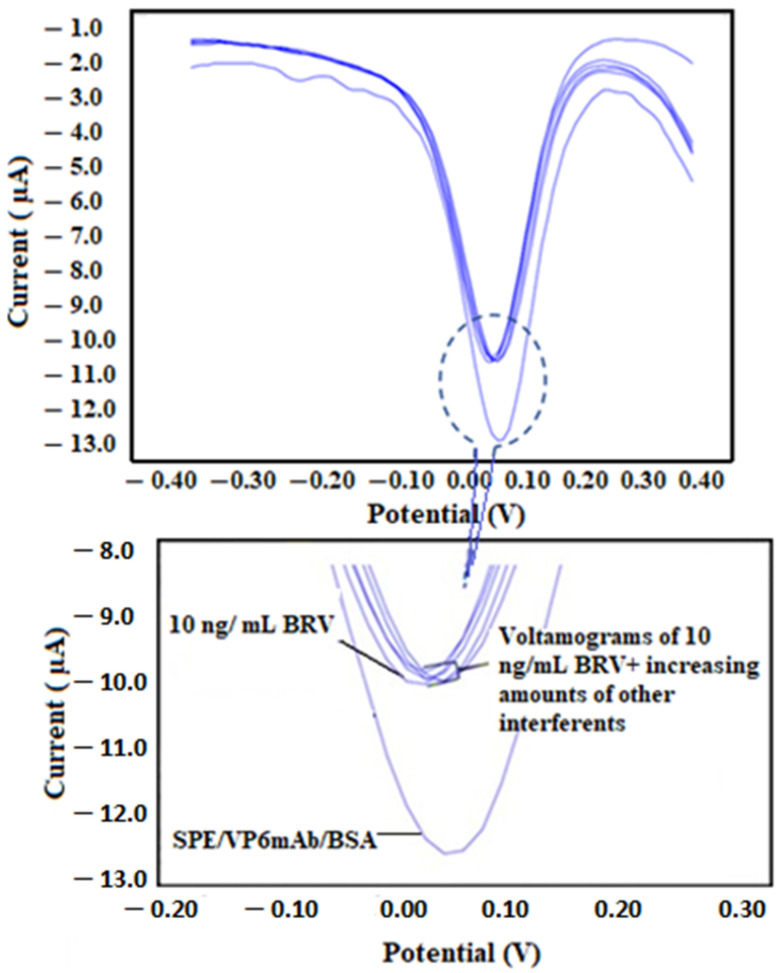
DPV voltammograms for the interference study. For selectivity experiments, the SPE/VP6mAb/BSA was tested in environments containing not only BRV but also BCoV, *E. coli* K99, and *C. parvum* parasite. For this purpose, 2, 5, 7, 10, and 15 ng/mL of each antigen were sequentially added to the environment containing 10 ng/mL BRV, and the resulting mixtures were applied to the SPE/VP6mAb/BSA immunosensor separately (*n* = 5).

For selectivity experiments, the SPE/VP6mAb/BSA was tested in environments containing not only BRV but also BCoV, *E. coli* K99, and *C. parvum* parasite. For this purpose, 2, 5, 7, 10, and 15 ng/mL of each antigen were sequentially added to the environment containing 10 ng/mL BRV, and the resulting mixtures were applied to the SPE/VP6mAb/BSA immunosensor separately. The responses obtained were compared with the current response from the condition without any interfering species. The results are shown in the DPV voltammograms in Figure 9. The percentage change in the current differences is presented in the bar chart in Figure S9. Additionally, the concentrations and selectivity/competition tests are detailed in Table 2.

**Figure 10:**
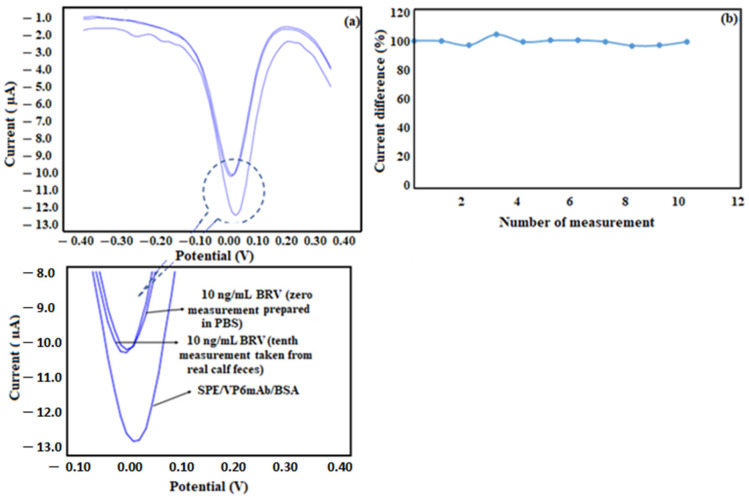
(**a**) DPV voltammograms of real sample analysis. (**b**) selectivity/competition percentage graph for real sample analysis (*n* = 10).

For the analysis of real samples, a healthy 3-year-old calf was chosen, and 5 g of this calf was taken by a sterilized sealed septum from an ordinary dairy plant. This feces sample was solubilized by PBS solution, then 10 ng/mL BRV was added to 1 mL of this solution to mimic the infected medium. Then the sample was applied to the surface of SPE/VP6mAb/BSA electrodes for 10 independent experiment cycles. The response obtained from the feces solution containing the same amount of BRV was compared with the current response obtained from the reference PBS solution containing 10 ng/mL BRV. The DPV voltammograms of the measurements are shown in Figure 10a, and the % selectivity/competition values are shown in Figure 10b. The % selectivity/competition value of 100 ± 2.95 was obtained for the real sample analysis (*n* = 10).

Finally, measurements were taken using a sandwich electrode with 10 ng/mL AuVP6mAb in PBS under optimal conditions, resulting in a selectivity/competition percentage of 100 ± 3.15.

### 3.7. Comparison of the Developed Immunosensors with Other Studies

The analytical performance of the developed MoS_2_/AuNP-based electrochemical immunosensor was compared with previously reported *Rotavirus* detection methods (Table 3). To the best of our knowledge, this study represents the first electrochemical biosensor specifically designed for *Rotavirus* VP6 protein detection using a dual-nanomaterial platform. Maeng et al. (2016) reported a hydrogel-based nanoporous photonic crystal biosensor for label-free *Rotavirus* antigen detection, achieving a limit of detection of 6.35 µg/mL (6350 ng/mL) [30]. Although their system offered label-free detection without sample pretreatment, our MoS_2_/AuNP sandwich immunosensor demonstrated approximately a 5700-fold improvement in sensitivity (1.11 ng/mL vs. 6350 ng/mL). This substantial enhancement is attributed to the synergistic effect of MoS_2_ nanoparticles, which provide high surface area and enhanced electron transfer, and AuNP-labeled secondary antibodies that amplify the electrochemical signal. Compared to conventional ELISA methods, which typically detect *Rotavirus* antigens at concentrations around 127 µg/mL (127,000 ng/mL) with analysis times of 2–3 h, our electrochemical platform offers over 100,000-fold higher sensitivity with significantly reduced analysis time. The developed immunosensor maintains excellent reproducibility (RSD = 1.89%, *n* = 5) and a wide linear range (0.05–10.0 ng/mL), making it suitable for quantitative *Rotavirus* detection in veterinary diagnostics. The targeting of VP6 protein—a highly conserved, group-specific antigen—enables pan-serotype detection of *Rotavirus* groups A, B, and C, providing broader diagnostic coverage than surface protein-based methods. These results demonstrate that the MoS_2_/AuNP electrochemical immunosensor offers superior sensitivity, rapid detection, and practical applicability compared to existing *Rotavirus* biosensing platforms, positioning it as a promising tool for point-of-care veterinary diagnostics.

## 4. Conclusions

This study successfully developed electrochemical immunosensors for the detection of BRV using both bare and MoS_2_ NP-modified SPEs. The modification with MoS_2_ NP significantly enhanced the electrochemical performance of the SPEs, improving both sensitivity and detection limits. Specifically, the MoS_2_ NP-modified immunosensor demonstrated an outstanding LOD of 0.95 ng/mL and LOQ of 3.18 ng/mL, with an RSD value of 5.83%, indicating good repeatability. The linear range for both immunosensors covered a wide spectrum, from 0.01 ng/mL to 10 ng/mL, with excellent correlation coefficients (R^2^ > 0.99), confirming their potential for sensitive and reliable virus detection. For the SPE with AuVP6mAb, the LOD was measured at 3.02 ng/mL, with an LOQ of 10.08 ng/mL. The RSD was calculated as 3.53% (*n* = 5), and the sensor exhibited a linear range of 0.01–10 ng/mL (R^2^ = 0.99). In the case of the MoS_2_-modified SPE with AuVP6mAb, the LOD was determined to be 1.11 ng/mL, while the LOQ was 3.72 ng/mL. The RSD for this electrode was calculated as 1.89% (*n* = 5), with a linear range of 0.05, 0.1, 0.5, and 10 ng/mL (R^2^ = 0.99).

The optimized immunosensor exhibited high selectivity, showing minimal interference from other pathogens such as *E. coli*, BCoV, and *C. parvum*. The real sample analysis using calf feces provided a selectivity/competition rate of 100 ± 2.95%, further supporting the applicability of the developed sensors in practical field conditions.

The findings of this study underline the significant advantages of electrochemical biosensors over traditional diagnostic methods, including ELISA and PCR, due to their cost-effectiveness, simplicity, and rapid response times. Moreover, the successful application of MoS_2_ NPs in the modification of the electrodes highlights the potential of nanomaterials in enhancing the sensitivity and performance of biosensors. This research paves the way for the development of a more versatile electrochemical diagnostic platform, with the potential to extend its application to other viral pathogens, contributing to the advancement of next-generation diagnostic tools.

## Figures and Tables

**Table 1:** Rct values for the semicircles of the Nyquist EIS spectra measured at different modification stages of the SPE-based immunosensor (*n* = 5).

Electrode	Rct Value
Bare SPE	228.37
SPE/VP6mAb	401.59
SPE/VP6mAb/BSA	478.37
SPE/VP6mAb/BSA/BRV	664.77
SPE/VP6mAb/BSA/BRV/AuVP6mAb	663.96

SPE: screen-printed electrode; VP6mAb: monoclonal anti-VP6 mAb antibodies; BSA: bovine serum albumin; BRV: *Bovine rotavirus*.

**Table 2:** Selectivity/competition test results and current values (*n* = 5).

Concentration of the Species *BcoV*, *E. coli* K99, and *C. parvum*	BRV Concentration	% Selectivity/Competition ± s
2 ng/mL	10 ng/mL	98.02 ± 0.37
5 ng/mL	10 ng/mL	99.74 ± 0.22
7 ng/mL	10 ng/mL	100.64 ± 0.36
10 ng/mL	10 ng/mL	99.21 ± 0.31
15 ng/mL	10 ng/mL	104.5 ± 0.37

**Table 3:** Comparison of the analytical performances of developed electrodes with previously reported studies.

Platform	Target	LOD	LOQ	RSD (%)	Linear Range	Time	Reference
SPE/VP6mAb/BSA/BRV	*Rotavirus*	1.66 ng/mL	5.53 ng/mL	3.49	0.01–10.0 ng/mL	30 s	This study
SPE/MoS_2_ NP/VP6mAb/BSA/BRV	*Rotavirus*	0.95 ng/mL	3.18 ng/mL	5.83	0.05–10.0 ng/mL	30 s	This study
SPE/VP6mAb/BSA/BRV (Au-VP6mAb labeled)	*Rotavirus*	3.02 ng/mL	10.08 ng/mL	3.53	0.01–10.0 ng/mL	30 s	This study
SPE/MoS_2_ NP/VP6mAb/BSA/BRV (Au-VP6mAb labeled)	*Rotavirus*	1.11 ng/mL	3.72 ng/mL	1.89	0.05–10.0 ng/mL	30 s	This study
Hydrogel based nanoporous photonic crystal	*Rotavirus*	6350 ng/mL	-	-	6350–1,270,000 ng/mL	30 min	[30]
ELISA	*Rotavirus*	127,000 ng/mL	-	-	-	2–3 h	[30]
Commercial ELISA	*Rotavirus*	1.000 ng/mL	-	-	-	2–3 h	Commercial ELISA kit for *Rotavirus* detection

ELISA: Enzyme-Linked Immunosorbent Assay; SPE: screen-printed electrode; VP6mAb; monoclonal anti-VP6 mAb antibodies; BSA: bovine serum albumin; Au-VP6mAb labeled: Gold nanoparticle-labeled antibodies.

## Data Availability

All relevant data generated or analyzed in this study are included in this article. The authors affirm that the data supporting the study’s findings are accessible within the publication.

## Supplementary Materials

The following supporting information can be downloaded at: https://www.mdpi.com/article/10.3390/life16030464/s1. Figure S1. Voltammograms of MoS_2_ NP quantity optimization on SPE electrodes. Figure S2. Optimization graph of MoS_2_ NP. Figure S3. Voltammograms corresponding to bindings performed at different pH values. Figure S4. Optimization graph of the pH optimization measurements. Figure S5. DPV voltammograms related to temperature optimization. Figure S6. Graph of Temperature Optimization. Figure S7. Optimization graph for incubation time. Figure S8. Optimization graph for incubation time. Figure S9. Recovery values obtained from interference measurements. Additional data including all detailed optimization curves (MoS_2_ concentration, pH, temperature, and incubation time) and interference study bar charts are provided in the Supplementary Information file.
