# Supplementary material for: Development of MoS2 Modified SPE Based Electrochemical Immunosensors Sandwiched by Au NP Labeled Antibodies for Detecting Bovine rotavirus in Calves
Source: Life (Basel). 2026 Mar 12;16(3):464. doi: 10.3390/life16030464 (PMC13027774; doi:10.3390/life16030464)
Supplement: Supplementary file 1 [file life-16-00464-s001.zip › life-4101409-supplementary.pdf]

SUPPLEMENTARY

Development of MOS_2 Modified SPE Based Electrochemical Immunosensors Sandwiched by Au NP Labeled Antibodies For Detecting *Bovine Rotavirus* in Calves

Ayşenur Akkaya[1], Derya Bal Altuntaş[2*], Aziz Kerim Çelik[1], Berkan Karagöz[1], Ümmünur Çelik[1], İbrahim Sözdutmaz[3], Sema Aslan[1], Ramin Jahangirov[4]

[1]Department of Chemistry, Mugla Sıtkı Kocman University, Muğla, Türkiye

[2]Department of Bioengineering, Faculty of Engineering and Architecture, Recep Tayyip Erdogan University, Rize 53100,Türkiye

[3]Department of Preclinical Sciences, Faculty of Veterinary Medicine, Erciyes University, Kayseri, Türkiye

[4]Department of Renewable Energy Sources and Technologies, Mugla Sıtkı Kocman University, Muğla, Türkiye

*Corresponding author: Derya Bal Altuntaş

E-mail : derya.balaltuntas@erdogan.edu.tr

1. Optimization of the Amount of MoS_2 NPs

Figure S1 shows the voltammograms for the quantity optimization of MoS_2 NPs added to SPE electrodes. At this stage, the DPV method was exclusively employed for optimization. The potential range for the responses from both bare and modified electrodes was set between -0.3 V and +0.3 V, with a scan rate of 50 mV/s.

In the quantity optimization process, the value for the 10 mg/mL suspension addition is not very precise because the MoS_2 NP overflows from the surface,

Figure S2. However, since the measurement was taken, it has been included in the graph.

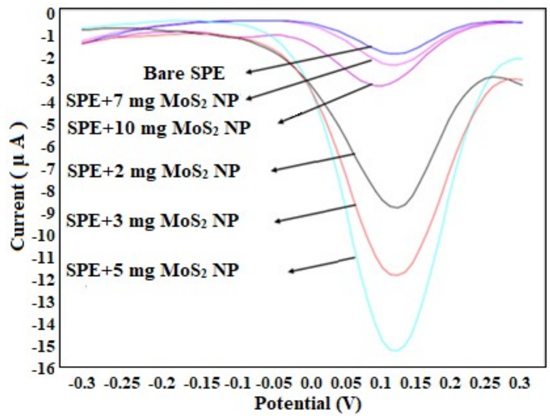

Figure S1. Voltammograms of MoS_2 NP quantity optimization on SPE electrodes.

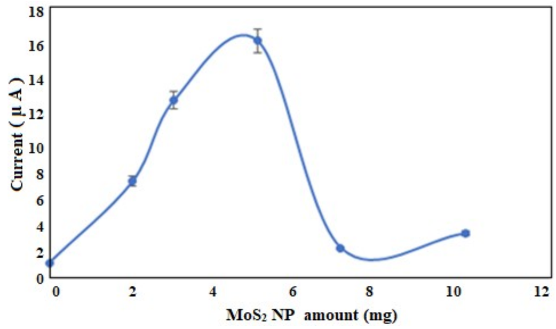

Figure S2. Optimization graph of MoS_2 NP.

2. pH optimization

To mimic conditions closest to the virus binding environment, PBS solutions with pH values ranging around 7.4, which is close to the physiological pH, were prepared. R solutions were prepared in these PBS solutions and applied onto the immunosensor, followed by DPV measurements. PBS solutions were prepared at pH values of 6, 6.5, 7, 7.5, and 8, and R solutions with a concentration of 10 ng/mL were prepared in Eppendorf tubes using these PBS solutions. The DPV graphs obtained from the measurements are shown in Figure S3, and the graph of the current values obtained from these voltammograms is provided in Figure S4.

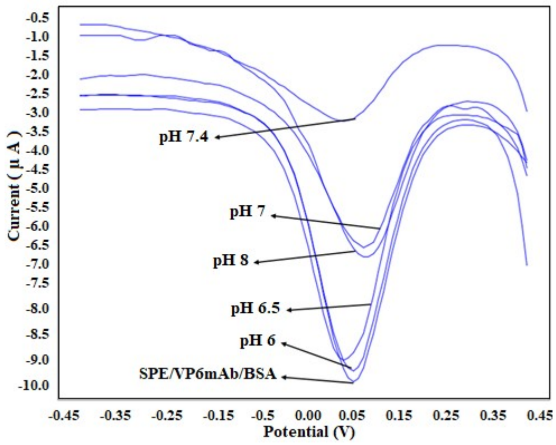

Figure S3. Voltammograms corresponding to bindings performed at different pH values.

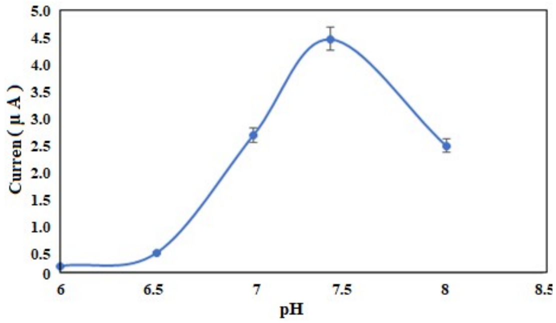

Figure S4. Optimization graph of the pH optimization measurements.

The best current difference obtained in the measurements was achieved at pH 7.4. This value was

determined as optimal and used in subsequent experiments.

3. Temperature Optimization

Similarly, to determine the optimal temperature, a temperature range around the physiological temperature of 36°C was selected, including 24, 28, 32, 36, and 40°C. PBS solutions at pH 7.4 were prepared and heated in water baths set to 24, 28, 32, 36, and 40°C, and 10 ng/mL R solutions were applied to the electrode surface at each temperature. The optimal binding temperature was determined based on these measurements. The DPV voltammograms obtained are shown in Figure S5, and the optimization graph derived from these voltammograms is presented in Figure S6. The results indicate that 36°C is the optimum temperature for the binding of the R antigen with the antibody.

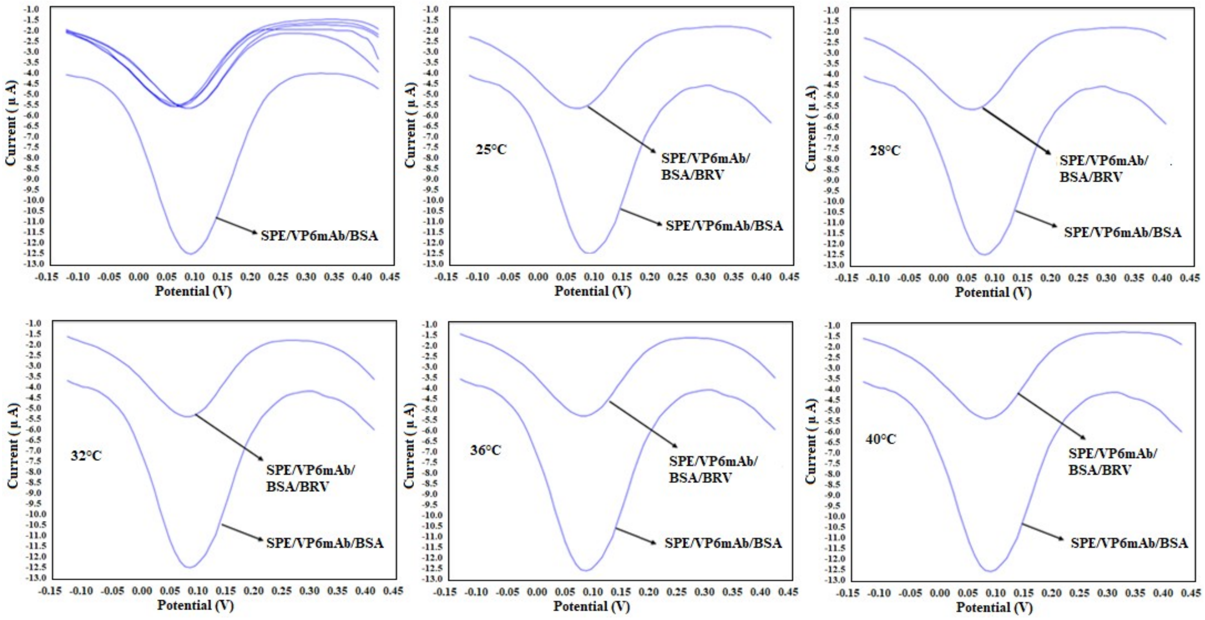

Figure S5. DPV voltammograms related to temperature optimization.

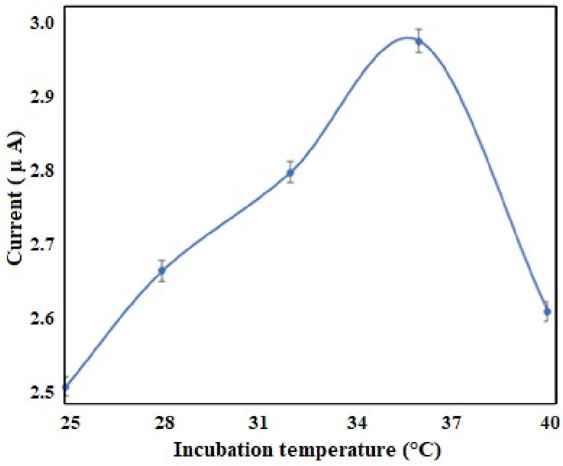

Figure S6. Graph of temperature optimization

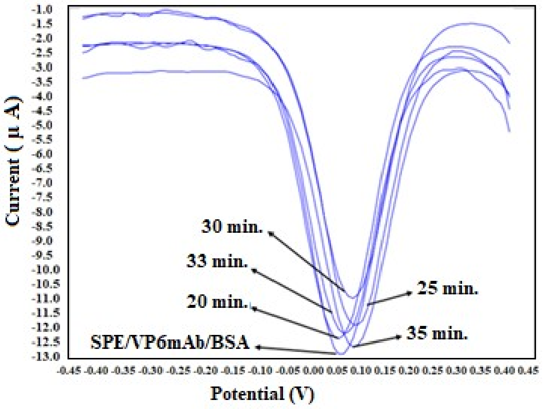

Figure S7. Optimization graph for incubation time.

4. Incubation Time Optimization

Finally, to determine the optimal incubation time, solutions at pH 7.4 and 36 °C were maintained, and the electrode was placed in a saturated vapor environment of this solution for incubation times of 20, 25, 30, 33, and 35 minutes. During this stage, the non-bound R antigens were washed off the electrode surface with PBS, and DPV measurements were taken.

The obtained voltammograms are shown in Figure S7, and the optimization graph derived from these voltammograms displaying current values is shown in Figure S8.

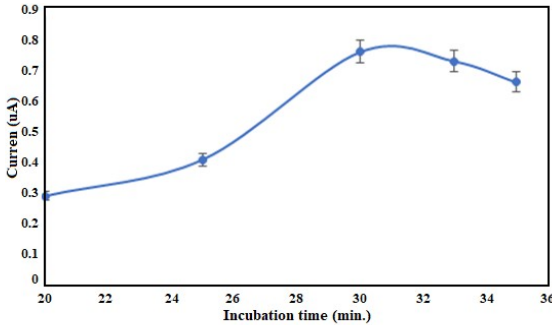

Figure S8. Optimization graph for incubation time.

At this stage, the optimum duration was determined to be 30 minutes. After this step, all optimizations were completed. The measurements performed using the optimum values are provided below.

5. Repeatability, Stability, Selectivity, and Real Sample Application Studies

The percentage change in the current differences is presented in the bar chart in Figure S9.

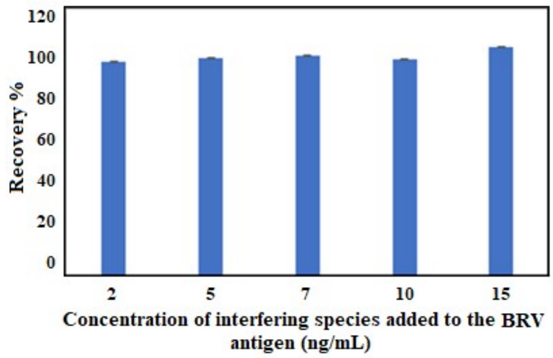

Figure S9. Recovery values obtained from interference measurements.
